# Shortness of Breath After Heart Surgery

**DOI:** 10.1016/j.acepjo.2024.100005

**Published:** 2025-01-10

**Authors:** Savannah Pocquette, Katelyn Levy, Jeffrey Gardecki

**Affiliations:** Department of Emergency Medicine, Jefferson Einstein Montgomery Hospital, East Norriton, Pennsylvania, USA

**Keywords:** complex pericardial effusion, cardiac tamponade, focused cardiac ultrasonography

## 1 Patient Presentation

A 56-year-old man with a history of aortic root aneurysm 3 weeks after surgery from an aortic root replacement with a mechanical valve-conduit presented to the emergency department with exertional dyspnea. He reported a week of progressive worsening of symptoms. He was afebrile with a heart rate of 112 cpm and blood pressure of 110/77 mm Hg. He had clear lungs and a faint systolic murmur without signs of volume overload. A point-of-care cardiac ultrasound was performed (Video 1). The ultrasound findings prompted consultation with cardiothoracic surgery.

**Video 1:**
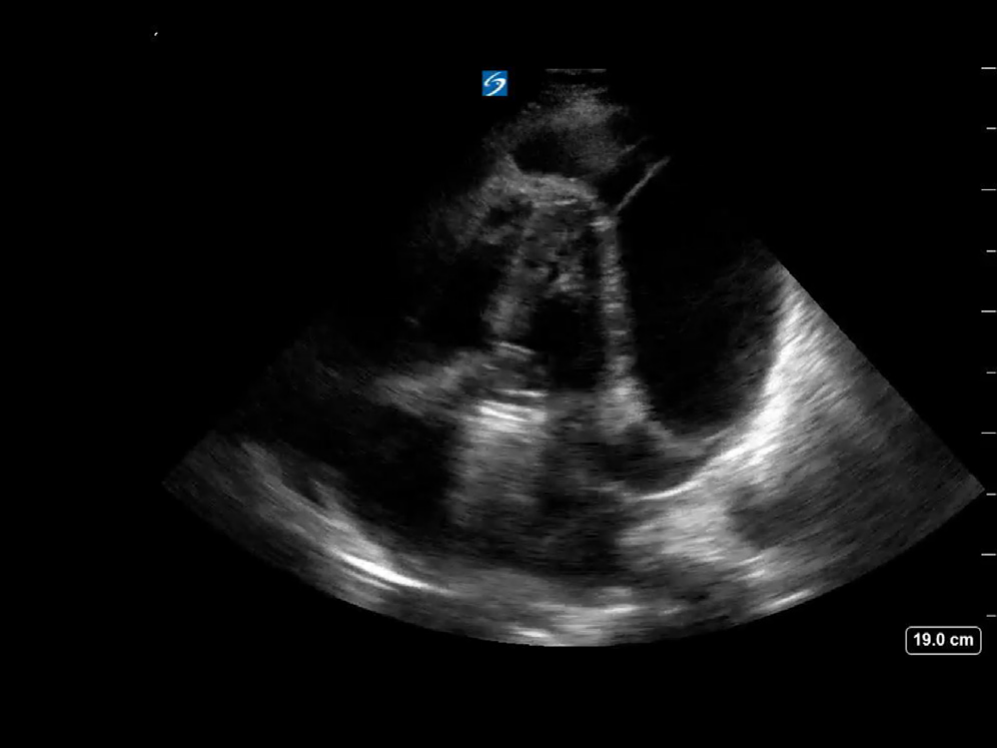
Apical 4 chamber view of the heart demonstrating a large circumferential complex pericardial effusion. Fibrin stranding is demonstrated beating with the atrioventricular valves. Signs of tamponade physiology are seen with both right ventricular and left ventricular collapse in diastole.

## 2 Diagnosis: Complex Pericardial Effusion Causing Tamponade

The patient was immediately taken to the operating room for a subxiphoid pericardial window for cardiac tamponade. Emergency physicians can use point-of-care ultrasound for the identification of pericardial effusions, which include states of profound shock and cardiac arrrest.^1^ The findings on point-of-care ultrasound that were suggestive of cardiac tamponade include the presence of a pericardial effusion with identification of cardiac chamber collapse (Fig).^2^^,^^3^ The specificity for tamponade goes up with progression of the chamber involved from the right atria and ventricle to eventually the left sided cardiac chambers.^3^ Collapse of the left atrium and ventricle can be seen in the apical 4 chamber clip. Postoperative tamponade can involve the left ventricle and is seen most frequently after valve surgery and in patients on anticoagulation.^3^^,^^4^

**Figure:**
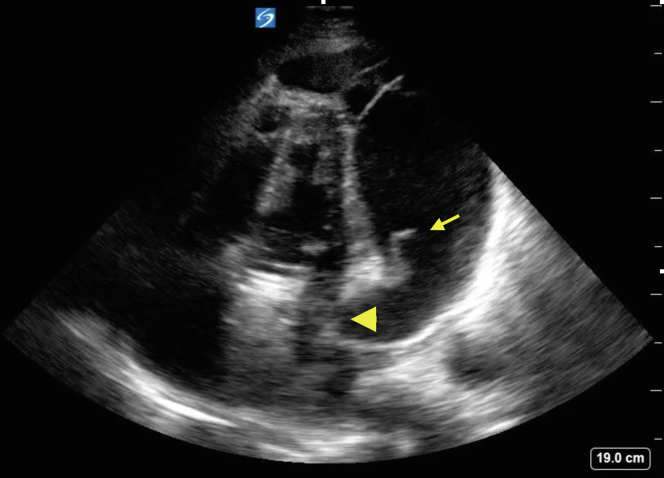
Fibrin strands (arrow) can be seen moving through the cardiac cycle mimicking the appearance of the atrioventricular valves. The right ventricle and atria are not well visualized on this image; however, diastolic collapse of the left atria (arrowhead) is evident.

## Conflict of Interest

All authors have affirmed they have no conflicts of interest to declare.
